# Tackling Anticancer Drug Resistance and Endosomal Escape in Aggressive Brain Tumors Using Bioelectronics

**DOI:** 10.1021/acsomega.4c05794

**Published:** 2024-10-08

**Authors:** Akhil Jain, Philippa Wade, Snow Stolnik, Alistair N. Hume, Ian D. Kerr, Beth Coyle, Frankie Rawson

**Affiliations:** † Division of Pharmacy and Optometry, School of Health Sciences, Faculty of Biology, Medicine and Health, University of Manchester, Oxford Road, Manchester M13 9PT, U.K.; ‡ Bioelectronics Laboratory, Division of Regenerative Medicine and Cellular Therapies, School of Pharmacy, Biodiscovery Institute, University of Nottingham, Nottingham NG7 2RD, U.K.; § Children’s Brain Tumour Research Centre, School of Medicine, Biodiscovery Institute, University of Nottingham, Nottingham NG7 2RD, U.K.; ∥ Division of Molecular Therapeutics and Formulation, School of Pharmacy, University of Nottingham, Nottingham NG7 2RD, U.K.; ⊥ School of Life Sciences, Queen’s Medical Centre, University of Nottingham, Nottingham NG7 2UH, U.K.

## Abstract

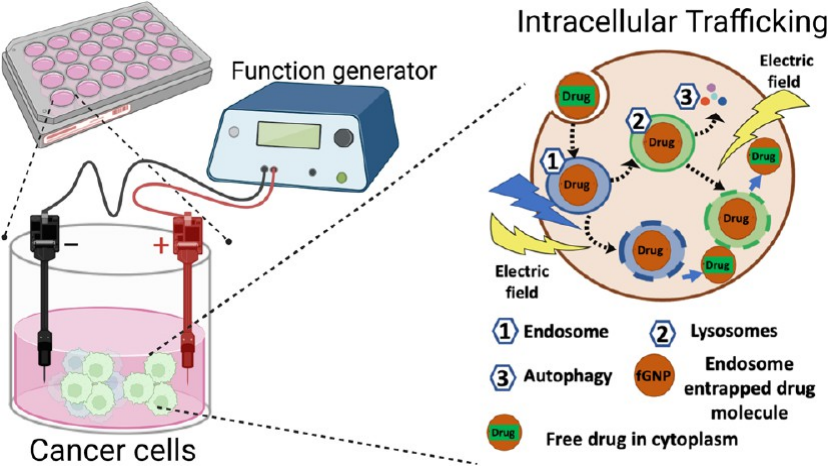

Resistance mechanisms in brain tumors, such as medulloblastoma and glioblastoma, frequently involve the entrapment of chemotherapeutic agents within endosomes and the extracellular expulsion of drugs. These barriers to effective treatment are exacerbated in nanotechnology-based drug delivery systems, where therapeutic nanoparticles often remain confined within endosomes, thus diminishing their therapeutic efficacy. Addressing this challenge necessitates the development of novel strategies to enhance the efficiency of cancer therapies. This study tests the hypothesis that external electrical stimuli can modulate intracellular trafficking of chemotherapeutic drugs in common malignant brain tumors in children (medulloblastoma) and adults (glioblastoma) by using gold nanoparticles (GNPs). In our experiments, alternating current (AC) stimulation ranging from 1 kHz to 5 MHz and at a strength of 1 V/cm significantly reduced cell viability in drug-resistant medulloblastoma and enhanced delivery of GNPs in glioblastoma. Low-frequency AC resulted in a 50% increase in apoptosis compared to controls and an 8-fold increase in cell death in cisplatin-resistant medulloblastoma cells, accompanied by a substantial reduction in EC_50_ from 2.5 to 0.3 μM. Similarly, vincristine-resistant cells demonstrated a 4-fold enhancement in drug sensitivity. Furthermore, high-frequency AC facilitated a significant increase from 20 to 75% in the endosomal escape of GNPs in glioblastoma cells. These findings underscore the potential of AC to selectively disrupt cancer cell resistance mechanisms and bolster the efficacy of nanoparticle-based therapies. The results indicate the effectiveness of AC stimulation in circumventing the limitations inherent in current nanotechnology-based drug delivery systems but also illustrates its transformative potential for treating aggressive, drug-resistant brain tumors.

## 1 Introduction

Cells have honed their ability to maintain homeostasis by delicately balancing their internal environment by exerting precise control over what enters and exits the cell. This fine-tuned regulation is primarily orchestrated by cell and organelle membranes, the gateway to the cell and organelles, respectively.^1^ The implications of this cellular control mechanism extend far beyond basic biological processes. They hold profound significance in the realm of disease treatment, particularly in the context of combating cancer. Cancer cells are notorious for their ability to evade therapeutic interventions and have evolved strategies to resist the effects of chemotherapy drugs through membrane-bound organelles and structures such as endosomes and extracellular vesicles (EVs), respectively, effectively thwarting their intended actions.^2^ This adaptation highlights the critical role played by cellular homeostasis in the development of resistance and underscores the need for novel approaches to tune these transport mechanisms.

EVs play a crucial role in the function of glioblastoma (GBM) and more broadly gliomas, which are difficult-to-treat cancers.^3,4^ Within this context, EVs have gained prominence due to their critical role in various cellular processes, including intercellular communication, cell signaling, and immune regulation.^5^ GBM cells secrete these small membranous vesicles, containing an array of cargo molecules such as proteins, RNA, and lipids.^6^ EVs play a significant role in GBM biology and pathogenesis, where they contribute to tumor growth, invasion, and metastasis by delivering oncogenic proteins and signaling molecules to other cells in the tumor microenvironment, suppressing the antitumor immune response, promoting angiogenesis and inducing resistance to chemotherapy and radiation therapy.^5^

EVs have also emerged as a crucial transport system in medulloblastoma. Medulloblastoma-derived EVs promote tumor growth, invasion, and metastasis, often by mechanisms akin to those observed in GBM-derived EVs.^7^ The therapeutic potential of EVs in GBM and medulloblastoma is under exploration. EVs could serve as vehicles for delivering therapeutic drugs or genes to tumor cells, modulating the tumor microenvironment to suppress tumor growth and progression, or developing vaccines targeting tumor-specific antigens.^8^ Furthermore, the role of EVs as biomarkers for monitoring disease progression and therapy response in patients with these aggressive brain tumors holds great promise.^9^ Importantly for this study, the reviews of the involvement of EVs in gliomas reveal evidence suggesting that glioma cells utilize the vesicles to expel therapeutics, thereby enhancing resistance.^4^ The challenges encountered in advanced drug delivery further underscore the importance of understanding cellular homeostasis.^10^ A long-standing challenge in advanced drug delivery is that drugs and nanoparticles can be trafficked and siloed in endosomes and subsequently degraded in lysosomes. To date, only a small fraction of these systems have advanced to clinical use, mainly due to issues such as entrapment in endosomes and degradation in lysosomes.^11^ For instance, delivery of siRNA and other chemotherapeutics by lipid nanoparticles or EV-based therapeutic delivery is limited by endosomal entrapment.^12,13^ This unfortunate fate can render potentially life-saving medications ineffective.^12^

Cancer cells significantly upregulate the release of EVs through several complex and interrelated mechanisms. Oncogenic mutations, such as those involving EGFRvIII, drive the production of EVs that transfer oncogenic factors to surrounding cells, enhancing tumor growth. The hypoxic conditions commonly found in tumors further stimulate EVs production by activating hypoxia-inducible factors, which promote angiogenesis and metastasis. Additionally, the tumor microenvironment itself, particularly through interactions with stromal cells, such as fibroblasts and endothelial cells, plays a crucial role in increasing EVs release. These stromal cells can influence the EVs production in cancer cells by secreting factors such as TGF-β, which can convert fibroblasts into myofibroblasts, enhancing local invasion and vascularization. Changes in cytoskeletal dynamics, driven by mutations in genes regulating actin and microtubules, also facilitate the release of EVs. Despite these advances, much remains to be understood about the specific molecular pathways and regulatory networks that control EVs biogenesis and release in cancer, highlighting the need for further research to explore these processes and their potential as therapeutic targets.

Consequently, there is an urgent need to develop widely applicable technologies that can modulate and precisely control the trafficking of drugs, molecules, and nanoparticles within cells. By unraveling the intricate mechanisms and developing disruptive technological approaches to govern intracellular transport, we can begin to overcome the challenges posed to drug delivery and enhance the efficacy of treatments. Such advancements hold promise for revolutionizing the field of medicine and could one day improve patient outcomes.

Bioelectronic medicines are an emerging therapeutic approach^14^ in which electrical input can be used for the treatment of disease. There has been a recent shift to develop a wireless electrical system for triggering the release of drugs from their carriers, as reviewed by Mirvakili and Langer.^15,16^ Additionally, electroporation has been heavily studied for enhancing cell delivery of an anticancer drug with the most recent study investigating this in single cells.^17^ We have previously demonstrated that ultrasound can be used to intracellularly release drug from liposomes.^18^ Our group has recently shown that AC could bypass the plasma membrane to exert effects across the cell membrane.^16,19,20^ Moreover, it has been established that electrical input on lipid bilayers causes subtle structural perturbations in their structure.^21^ Therefore, we hypothesized that by exploiting electrical input, intracellular fate of nanoparticle-based delivery systems for anticancer therapy could be modulated. This could influence two distinct processes (i) in cancer treatment: transport of anticancer drug out of cell via EVs; moderating EVs transport processes would lead to increased cytoplasmic exposure of the drug and cancer cells killing, and (ii) release of GNPs from endosomal/lysosomal compartment into cytoplasm. This could lead to the generation of a new nanomedicine for improved therapeutic outcomes. To the best of our knowledge, there has been no demonstration of using AC to prevent the transport of chemotherapies outside of cells via EVs. Furthermore, the added potential of using AC would increase the drug delivery system’s presence within the cytoplasm by facilitating endosomal escape.

**Scheme 1:**
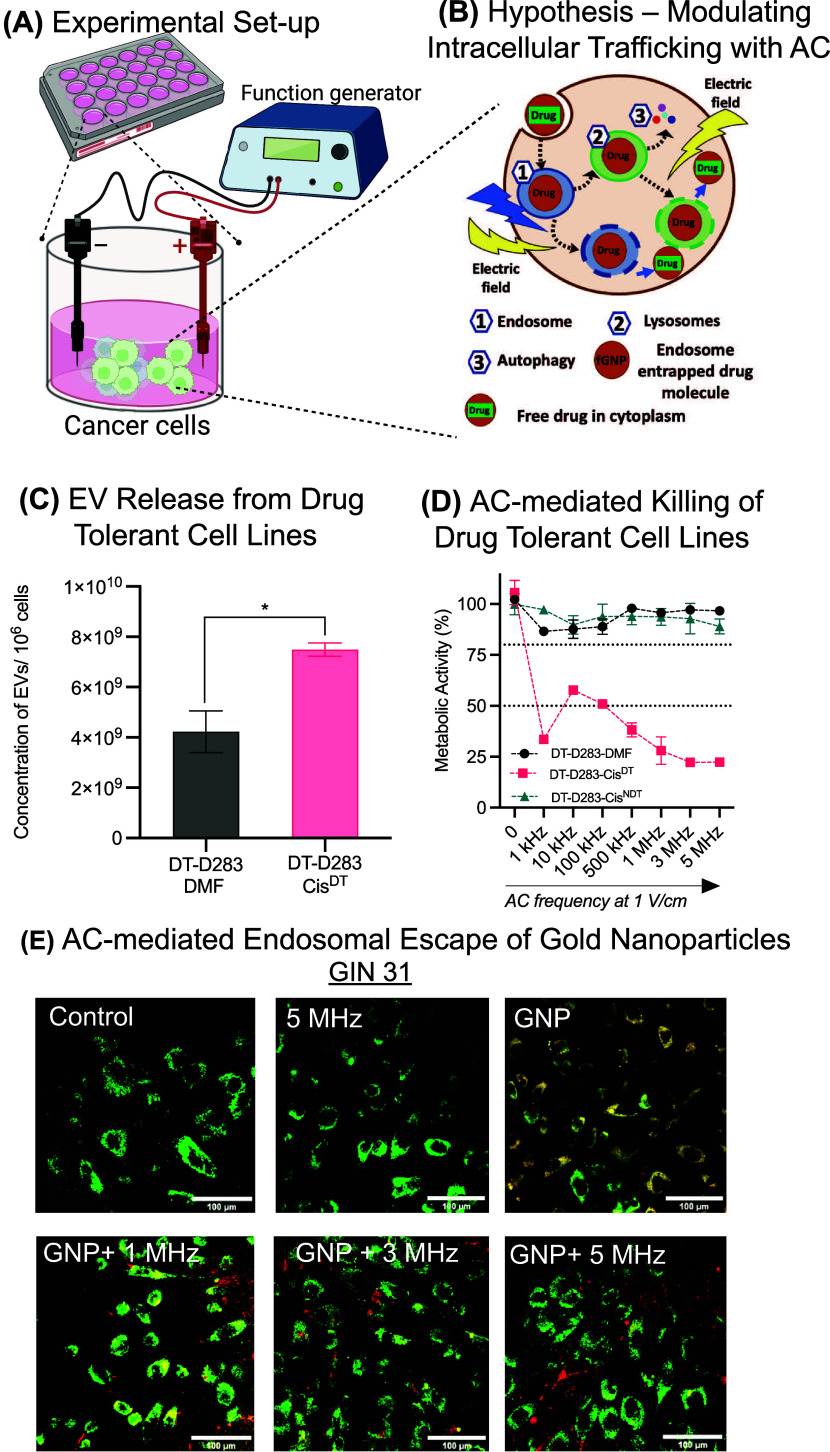
Schematic Representation of Experimental Setup and Methods of Tackling Anticancer Drug Resistance and Endosomal Escape in Aggressive Brain Tumors Using Bioelectronics (A) Schematic representation of the experimental setup used for the study. This setup was designed to investigate the effects of different frequencies on chemo-resistant cancer cells when exposed to gold nanoparticles (GNPs). (B) Hypothesized mechanism illustrating brain tumor cells when exposed to varying AC frequencies may enhance the delivery of GNPs and efficacy of chemotherapeutic agents by inducing cellular stress and promoting nanoparticle-endosome release. (C) Zeta view analysis of EVs showing their distribution within the cellular environment. This analysis is crucial for understanding the interaction between EVs release and drug resistance. (D) Cell viability assay results, demonstrating the effects of different AC frequencies (1, 3, and 5 MHz) at 1 V/cm on the viability of chemo-resistant cancer cells. The data highlights the AC frequency-dependent response of the cells, with a particular focus on the enhancement of therapeutic outcomes at specific frequencies. (E) Confocal microscopy images illustrating the correlation between GNPs and endosomes within the GIN 31 cells. These images provide visual evidence of the intracellular localization and potential mechanisms by which GNPs influence cellular processes, particularly in the context of enhancing the delivery of chemotherapeutic agents.

In this work, we conducted a study aiming to merge electronics for the delivery of AC with chemotherapy to chemoresistant (cisplatin-cis and vincristine-vin) medulloblastoma and GBM cells. Our objective was to investigate whether this delivery of high-frequency-AC (HF-AC) to cells could help overcome the problems of (i) EV-mediated drug transport out of cancer cells and (ii) modulate the intracellular escape of GNPs from endosomes by influencing the transport process. The results of our study successfully demonstrate this concept, indicating that by combining electronics with anticancer therapy, we can potentially introduce a new bioelectronic technology that holds promise for the treatment of resistant cancers. Further development of this approach could enhance the efficacy of chemotherapies and, more broadly, the field of cancer therapeutics. A diagrammatic representation of the setup can be seen in Scheme 1.

## 2 Methodology

### 2.1 Cell Lines and Standard Culture Conditions

Nomenclature and identifiers (chemotherapeutics agents are cisplatin (Cis) and vincristine (Vin)) of the cell lines used in this work are as follows: DT = drug tolerant, ^DT^ = drug treated, and ^NDT^= non-drug treated. For example, DT-XXX-Cis/Vin^DT^ means drug tolerant (DT) XXX cell line, tolerant to cis or vin, and are drug treated (^DT^). Similarly, DT-XXX-Cis/Vin^NDT^ means drug tolerant (DT) XXX cell line, tolerant to cis or vin, and is non-drug treated (^NDT^). Vehicle cell line is matched DMSO or DMF and generated alongside to account for morphological and genetic changes resulting from vehicle (DMF or DMSO) exposure and long-term culture. For example, DT-XXX-DMSO/DMF = Drug tolerant (DT) XXX vehicle cell line grown in cell culture medium containing equivalent volume of DMSO or DMF that has been used in drug-treated tolerant lines.

Sonic-hedgehog (SHH) medulloblastoma DAOY cell line was purchased from ATCC (ATCC HTB-186) and grown in DMEM with 10% fetal bovine serum (FBS, HyClone (Logan, Utah)). Three vehicle and corresponding cis-tolerant MB Group3 cell lines (DT-D283-DMF, DT-D283-Cis, DT-HD-MB03-DMF, DT-HD-MB03-Cis, DT-D458-DMF, and DT-D458-Cis) were utilized, as previously published.^22^ The DT-D283 and DT-D458 lines were derived in-house, whereas the DT-HD-MB03-Cis was obtained from Gianpiero Di Leva (Keele University, U.K.). DT-D283 and DT-D458 cells were cultured in DMEM with 10% fetal bovine serum and the DT-D283-Cis^DT^ and DT-D458-Cis^DT^ culture was supplemented with 1.6 and 0.6 μM cis (Selleckchem, Houston, TX, S1166), respectively. DT-HD-MB03 were grown in RPMI 1640 with 10% FBS and the DT-HD-MB03-Cis^DT^ cells were supplemented with 0.5 μm of cis. The equivalent volume of vehicle (DMF) was added to the matched vehicle line. All cell lines were mycoplasma tested monthly and grown in antibiotic-free culture conditions at 5% CO_2_ and 37 °C. EV-depleted FBS was generated by ultracentrifugation at 100,000 *g* at 4 °C for 18 h. Filter sterilization using a 0.22 μm filter (Millipore) was carried out prior to its addition to culture medium, resulting in an EV-depleted medium. GBM cells–Glioma INvasive Marginal 31 (GIN) cells from the infiltrative tumor margin and Glioma Core Enhanced 31 (GCE) from the core of the tumor were isolated previously.^23^ For studying endosomal escape of GNPs, both GIN and GCE cells were cultured in DMEM (Gibco) supplemented with 10% FBS, 1% penicillin/streptomycin, and 1% l-glutamine. Cells were maintained at 37 °C in an incubator containing 5% CO_2_. Cells were tested for mycoplasma every month, where they were grown in an antibiotic-free medium for 1 week before mycoplasma testing. All cells used were mycoplasma-free.

### 2.2 Generation of Vin- and Cis-Resistant Cell Lines

A continuous model of selection was used to generate drug-tolerant MB cell lines resistant to vis and cis. SHH DAOY cells were cultured continuously in the presence of vin (Selleckchem, S1241) and the concentration dose was escalated upon cell proliferation. Cells were passaged in T-25 flasks and initially treated with 1/10th of their vin EC_50_, and the dose increased upon cell proliferation. A matched DMSO vehicle cell line was generated alongside to account for morphological and genetic changes resulting from vehicle exposure and long-term culture. Cells were considered resistant when the EC_50_ against vin for the treated cells had exceeded the treatment dose and was significantly increased in comparison to the EC_50_ of the vehicle line. DT-DAOY cells were grown in DMEM with 10% FBS, and the DT-DAOY-Vin^DT^ line was supplemented with 2.8 nM vin. Akin to the DT-DAOY cells, cis-resistant cell lines DT-D458, DT-HD-MB03, and DT-D283 were also generated in a similar manner.^22^ Here, cells were treated with increasing doses of cis until their EC_50_ exceeded the treatment dose or was significantly increased in comparison to the vehicle cell line (DMF).

### 2.3 Drug Cytotoxicity Assay

Cells were seeded into clear-bottom, black-walled 96-well plates (Greiner; 655096) at a density of either 1 × 10^3^ (DT-DAOY), 5 × 10^3^ (DT-HD-MB03), or 1 × 10^4^ (DT-D283 and DT-D458) cells per well and left overnight. Cells were then challenged with varying concentrations of vin or cis prior to being incubated for 72 h at 37 °C and 5% CO_2_. After 72 h, metabolic activity was assessed using PrestoBlue (Thermo Fisher; A13262), and fluorescence was measured using the FLUOstar Omega microplate reader at 560/590 nm. Cell viability was calculated as a percentage relative to the vehicle control, and EC_50_ was calculated in GraphPad PRISM 9 using nonlinear regression with three parameters. Significant differences between EC_50_ were calculated using one-way ANOVA with Sidak’s multiple comparisons. The data represent the SEM of three independent experiments.

### 2.4 Isolation of Extracellular Vesicles

To isolate EVs from cell cultures, cells were grown in a 1× T-225 flask up to 30% confluence, washed twice with Hanks’ Balanced Salt Solution (HBSS, Gibco), and incubated in EV-depleted medium for 48 h. Cell culture medium was collected and centrifuged at 300*g* for 5 min to pellet cells. The supernatant was then centrifuged again at 1500 *g* for 10 min, followed by a final centrifugation at 10,000 *g* and 4 °C for 10 min to remove any debris and large particles. The supernatant was filtered through a 0.22 μm filter prior to ultrafiltration using a 100 K MWCO protein concentrator (Thermo Scientific Pierce; 88533), where the supernatant was centrifuged at 3000 *g* until an ∼1 mL concentrate was left. The 1 mL concentrate was loaded directly onto size-exclusion chromatography columns obtained from Izon (qEV1/70 nm; IC1-70) and EV fractions were collected according to the manufacturer’s instructions. The concentration of EVs was determined using ZetaView Nanoparticle Tracking Analysis relative to 1 × 10^6^ cells.

### 2.5 AC Stimulation

Electrical stimulation (ES) with AC was carried out by inserting two steel electrodes (0.5 × 25 mm^2^) at the opposite end (fixed at 10 mm from each other) of each well in a 24-well plate and dipped in cell culture medium. These electrodes were connected to an Arbitrary Function Generator (AFG-21225, RS PRO, U.K.) that delivered the AC sine-wave signals, frequency, and amplitude. The cells were stimulated with AC with a desired frequency and peak voltage amplitude of 1 V/cm for 30 min. The strength of AC between the electrodes was measured by using a digital oscilloscope (TDS 210, Tektronix).

### 2.6 Metabolic Activity Assay

The medulloblastoma vehicle cell lines DT-D283-DMF, DT-D458-DMF, DT-HD-MB03-DMF, and DT-DAOY-DMSO and the cell lines resistant to cis-drug treated (DT-D283-Cis^DT^, DT-D458-Cis^DT^, and DT-HD-MB03-Cis^DT^) and non-drug treated (DT-D283-Cis^NDT^, DT-D458-Cis^NDT^, and DT-HD-MB03-Cis^NDT^) and vin-drug treated (DT-DAOY-Vin^DT^) and non-drug treated (DT-DAOY-Vin^NDT^) were seeded at density of 1.0 × 10^5^ per well in a 24-well plate. The cells were stimulated with AC using the protocol mentioned in Section 2.5. Immediately after stimulation with AC, the cells were incubated at 37 °C and 5% CO_2_ for 24 h before carrying out the metabolic activity assay. Next, the medium containing cells was replaced with fresh medium containing 1% PrestoBlue HS cell viability reagent (Thermo Fisher Scientific, U.K.) and incubated for 1 h before reading the fluorescence at 590/610 nm (excitation/emission) in a microplate reader (Tecan Infinite M Plex and Spark 10M). Cells grown in culture medium without AC treatment were used as the negative control. Results are presented relative to negative control. The data is represented as an average of triplicate experiment with 3 independent repeats ± SEM.

### 2.7 Live/Dead Assay

After stimulation with AC, the cells were incubated for 24 h in an incubator at 37 °C and 5% CO_2_. Next, Cis-resistant and vehicle cells were centrifuged at 300*g* for 5 min (due to their semiadherent nature), and the pellet was dispersed in fresh medium containing 1 mM calcein AM and 1 mg/mL propidium iodide (Thermo Fisher, U.K.), and incubated for 30 min at 37 °C and 5% CO_2_ in a 24-well plate. The cells were then centrifuged (300*g* for 5 min), and the cell pellet was washed with PBS. Finally, the cells were placed in a 24-well plate (m-plate 24-well black, ibiTreat, Thistle Scientific, U.K.) in phenol red-free medium and imaged using a Leica TCS SPE confocal microscope. The proportions of live and dead cells were quantified using ImageJ software.

### 2.8 Biocompatibility of GNPs

The GIN and GCE cells were seeded in a 96-well plate at a density of 5 × 10^3^ cells/well and allowed to adhere for 24 h. The medium was replaced with fresh medium containing Texas red conjugated 100 nm spherical gold nanoparticle (GNP, Nanopartz, Inc.) at different concentrations (25, 50, and 100 mg/mL), and the cells were incubated for 8 h. Cells with nanoparticle suspensions were then stimulated with AC of various frequencies at 1 V/cm for 30 min. Following stimulation cells were then incubated for 24 h at 37 °C and 5% CO_2_. Next, the medium was replaced with complete medium containing 10% PrestoBlue HS cell viability reagent (Thermo Fisher Scientific, U.K.) and incubated for 1 h before reading the fluorescence at 590/610 nm (excitation/emission) in a microplate reader (Tecan Infinite M Plex and Spark 10M). The data are represented as an average of triplicate experiment with 3 independent repeats.

### 2.9 Endo/Lysosomal Escape

GIN 31 and GCE 31 cells were seeded at a density of 4 × 10^4^ cells per well in a 24-well plate and incubated at 37 °C and 5% CO_2_. After 24 h, the culture medium was replaced with fresh medium containing CellLight Late Endosomes-GFP, BacMam 2.0 (Thermo Fisher Scientific, U.K.) and incubated overnight at 37 °C and 5% CO_2_. Later, the medium was replaced with fresh medium containing 25 mg/mL GNP (in PBS) and the mixture was incubated for 8 h. Next, (AC at varying frequencies ranging from 1 kHz to 5 MHz) wasd applied for 30 minutes at 1 V/cm. Immediately after the AC stimulation, the cells were washed with PBS and imaged by using a Leica TCS SPE confocal microscope.

## 3 Results and Discussion

### 3.1 Drug Resistance in Medulloblastomas

**Figure 1:**
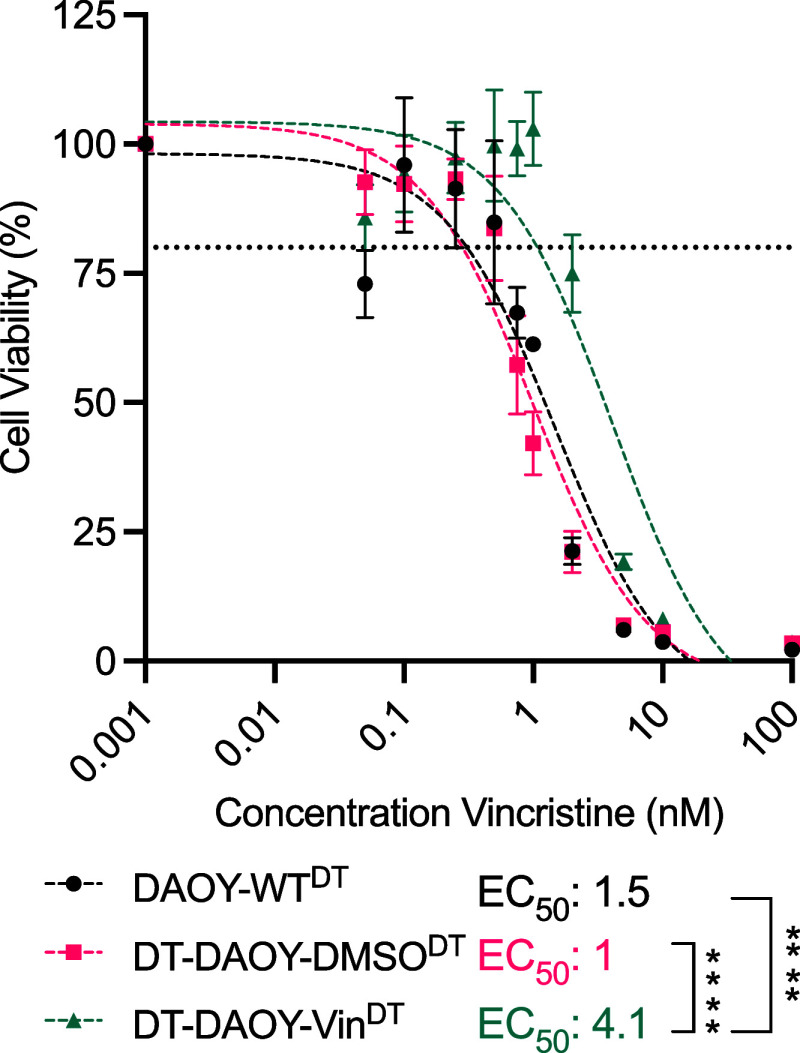
Continuous long-term vin treatment promotes increased cell resistance to vin in medulloblastoma SHH DAOY cells. The cells were initially treated with vin at 1/10th of their EC_50_ and upon cell proliferation, and the cells were subsequently challenged with an increasing dose of vin. Cell viability was assessed via drug-response assays, and the EC_50_ was calculated using nonlinear regression analysis with three parameters. For a detailed description of the cell lines and their identifiers used in this work, please refer to Section 2.1. DAOY-WT^DT^ = Wild-type DAOY cell line treated with Vin, DT-DAOY-Vin^DT^ = Vin tolerant cell line treated with Vin, and DT-DAOY-DMSO^DT^ = Vehicle cell line treated with Vin. Significance was assessed using one-way ANOVA with Šídák’s multiple comparisons test. ****p ≤ 0.0001.

Cis and vin are two of the standard chemotherapies for medulloblastoma. Previously we have described three cis-resistant medulloblastoma cell lines;^22^ in that work, compared to matched vehicle controls, DT-D458-Cis^DT^ showed 18.5-fold resistance, DT-HB-MB03-Cis^DT^ showed 1.6-fold resistance, and DT-D283-Cis^DT^ showed 2.5-fold resistance (for cell line identifier, please refer to Section 2.1). In the current work (Figure 1), we established a vin-tolerant DAOY cell line (DT-DAOY-Vin^DT^) by continuous treatment of cell culture with escalating concentrations of vin. DT-DAOY-Vin^DT^ has a 4-fold increase in the EC_50_ compared to the matched vehicle cell line (DT-DAOY-DMSO) (*p* < 0.001, Figure 1).

**Figure 2:**
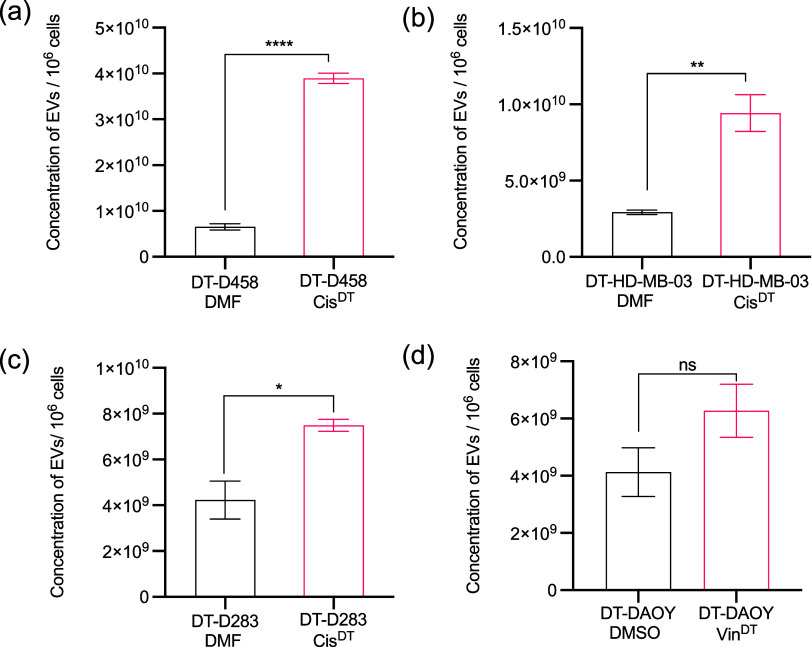
Quantification of EVs released in drug-tolerant cell lines. (a–c) Cis-tolerant MB_Group3_ cells release significantly more EVs in comparison to their matched vehicle cell lines. For a detailed description of the cell lines and their identifiers used in this work, please refer to Section 2.1. Cis-resistant and cis-treated cell lines are denoted as DT-D458-Cis^DT^, DT-D283-Cis^DT^, DT-HD-MB03-Cis^DT^, and vehicle cell lines are denoted as DT-D458-DMF, DT-D283-DMF, and DT-HD-MB03-DMF line. (d) EVs release from vin-tolerant DAOY cells. Vin-resistant and vin-treated cell lines (DT-DAOY-Vin^DT^) and vehicle (DT-DAOY-DMSO) cell lines. EVs in both cis- and vin-tolerant/vehicle cell lines were isolated using size-exclusion chromatography, and particle concentration from the pooled EVs-containing fractions was quantified using ZetaView nanoparticle tracking analysis. The concentration of EVs was calculated relative to 1 × 10^6^ cells, where cells were counted at the point of harvest. Significant differences between vehicles and cis/vin-tolerant cells were calculated using an unpaired *t* test. The data represent the average ± SEM of three independent experiments. *p ≤ 0.05, **p ≤ 0.01, and ****p ≤ 0.0001.

Intriguingly, a comparison of the number of EVs released revealed a significant increase by cis-tolerant lines (Figure 2a–c) relative to their vehicle counterparts (Figure 2). On the other hand, in vin-tolerant lines, no significant difference was observed compared to vehicle cells (Figure 2d). Nevertheless, some increase in the average number of EVs in drug-treated vin-tolerant lines was observed, which can be further supported by the literature, which also indicated that EVs can act as transporters for efflux of drugs.^24^ Thus, it can be concluded that the number of EVs released is enhanced in drug-tolerant lines, which together with EC_50_ data suggests that EVs mediated drug tolerance in medulloblastoma cell lines.

### 3.2 Overcoming EVs-Mediated Cis and Vin Resistance in Medulloblastoma

**Figure 3:**
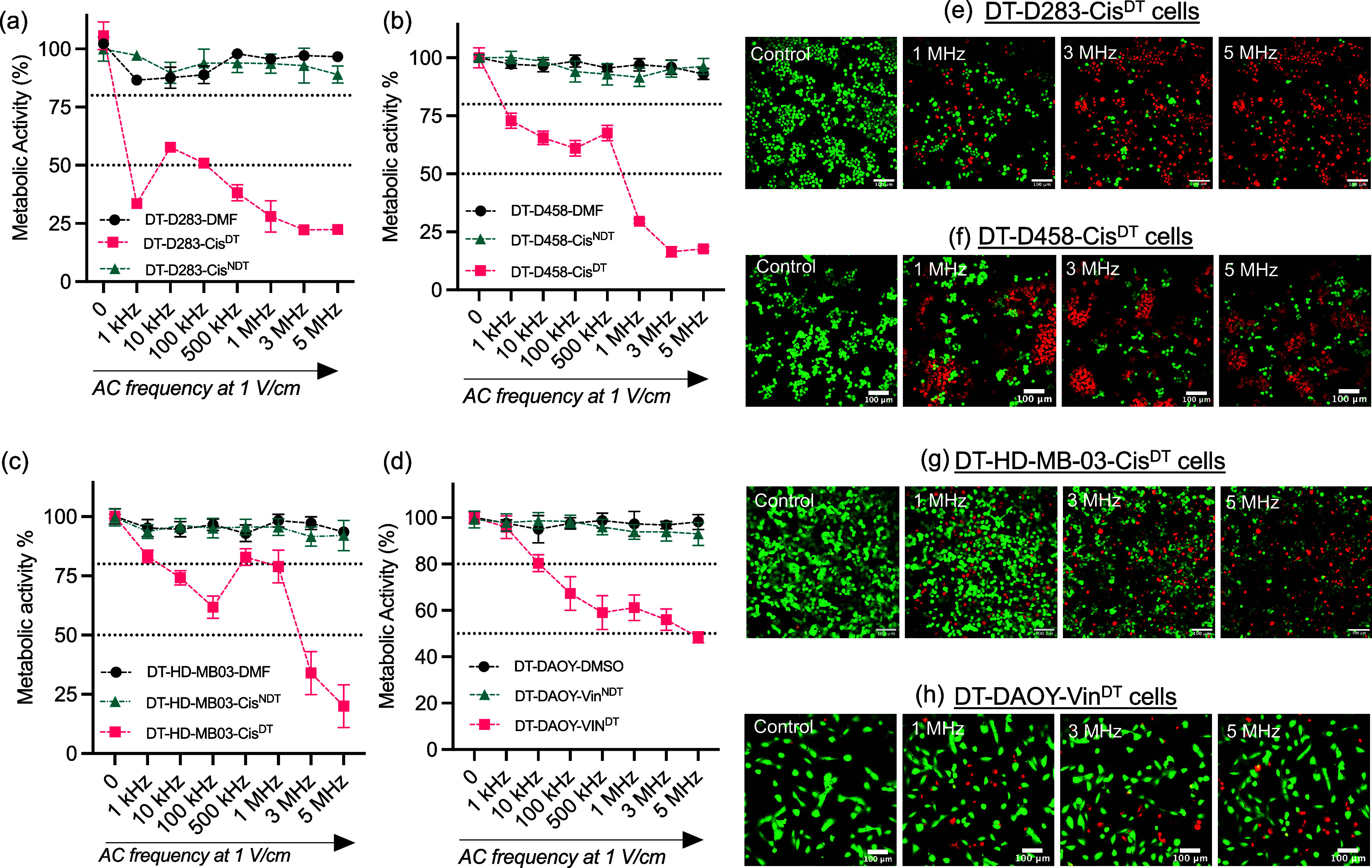
AC treatment overcomes cis and vin resistance in medulloblastoma cells *in vitro*. The cells were stimulated with sinewave AC (1, 3, and 5 MHz) using a frequency generator at a potential of 1 V/cm for 30 min followed by a PrestoBlue assay 24 h post electrical stimulation. For a detailed description of the cell lines and their identifiers used in this work, please refer to Section 2.1. (a–c) Metabolic activity of cis resistant cells treated with cis (DT-D283-Cis^DT^, DT-D458-Cis^DT^, DT-HD-MB03-Cis^DT^), cis-resistant but non-cis-treated cell lines (DT-D283-Cis^NDT^, DT-D458-Cis^NDT^, DT-HD-MB03-Cis^NDT^), and vehicle cell lines (DT-D283-DMF, DT-D458-DMF, DT-HD-MB03-DMF) cell line. (d) Vin-resistant cells treated with vin (DT-DAOY-Vin^DT^), vin resistant but non-vin-treated cell lines (DT-DAOY-Vin^NDT^), and vehicle (DT-DAOY-DMSO) cell lines. ^NDT^ = no drug treatment, and ^DT^ = drug treated. The error bars represent SEM of three independent experiments in triplicates. (e–h) Live/dead staining of cis and vin resistant medulloblastoma cell lines. The cells were stained with calcein AM (green, live cells) and propidium iodide (red, dead cells) 24 h after stimulation with AC-EFs and imaged using GFP and a Texas red filter in a Leica TCS SPE confocal microscope. Scale bars: 100 μm.

After establishing EVs-mediated drug resistance in medulloblastoma cell lines, we sought to provide evidence to this effect and investigate whether bioelectronics systems can be used to deliver electrical input, which modulates the efflux of drug through this pathway. To study the response to external electrical input on the manipulation of intracellular trafficking modulated by subcellular entities such as EVs, we used AC (Figure 3). AC with a frequency range of 1 kHz to 5 MHz at a constant potential of 1 V/cm was utilized to study the response. It is worth emphasizing that the AC used in this work are not tumor-treating fields, as the electrodes were not shielded by dielectric material, and the applied AC does not cause any significant change in the temperature of cell culture medium.^16^ To avoid electrolysis of cell culture, medium potentials above 1 V/cm were avoided. For all 4 cell lines treated, a decrease in metabolic activity was observed only in the drug-tolerant line (treated with cis or vin) when an AC was applied (Figures 3a–d and S1). This effect increased at higher frequencies (Figure 3a–d), which was confirmed to be cell death caused by enhanced concentration of either cis or vin within the cytoplasm (Figure 3e–h), as the drug-tolerant cell lines that were not treated with cis/vin did not show any cell death (Figure S2). This was fitting with previous findings that outer membranes are capacitively coupled to AC at high frequency (kHz–MHz). This leads to membrane electro-permeabilization, which further allows the effect of HF-AC on subcellular structures.^25^ Furthermore, no change in the viability of control cells (not drug treated) after the treatment with HF-AC suggests the interaction of HF-AC with membrane-bound EVs carrying anticancer drugs. This could be supported by previous reports in the literature where low-voltage electric fields lead to the disruption of EVs and release of their content.^26^ Therefore, based on the obtained data, it can be concluded that HF-AC could manipulate intracellular drug trafficking by affecting membrane-bound EVs, which leads to increased vulnerability of resistant cells toward cis and vin. The control experiments previously performed by us show that there are no significant effects of high-frequency AC on nontarget cells, suggesting that the method is selectively effective against cancer cells without causing unintended damage to healthy cells. This indicates that the use of high-frequency AC for the transport of nanoparticles carrying drugs for delivery could be safe, at least under the tested experimental conditions.^16^

### 3.3 Endo/Lysosomal Escape of Gold Nanoparticles

**Figure 4:**
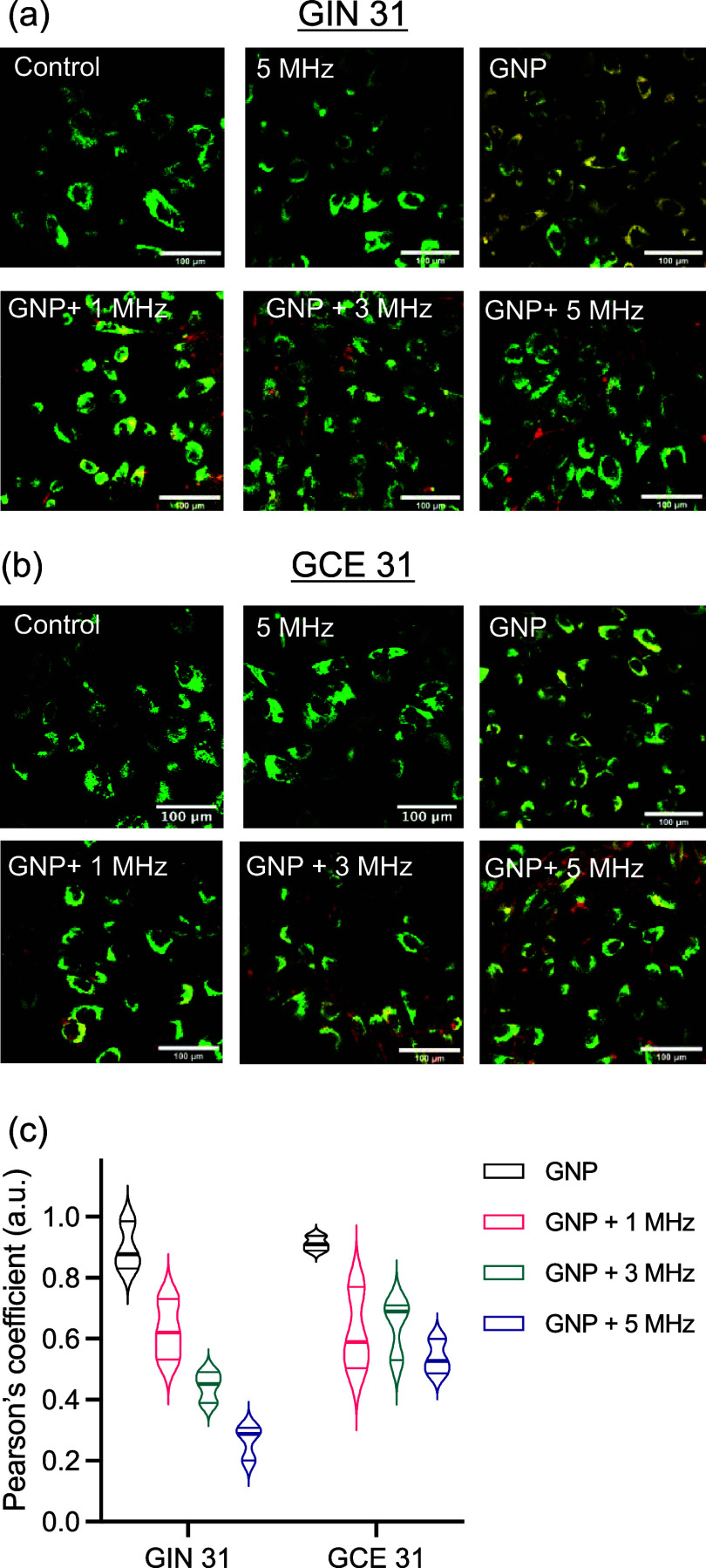
AC-mediated endosomal escape of GNPs in patient-derived glioblastoma cells (GIN 31 and GCE 31). Confocal microscopy images to demonstrate endosomal escape of GNPs (8-h incubation with cells) in (a) GIN 31 and (b) GCE 31 cells immediately after treatment with sine wave HF-AC (3 and 5 MHz) at a potential of 1 V/cm for 30 min. Cells were stained with late endosome dye (green) and imaged by using a Leica confocal microscope with GFP (late endosomes) and Texas-red (GNPs) filter settings. Scale bar: 100 μm. For a detailed description of the cell lines and their identifiers used in this work, please refer to Section 2.1. To confirm the colocalization of GNPs, at least 50 cells were analyzed. (c) Violin plot depicting Pearson’s correlation coefficient (obtained using colocalization plugin in ImageJ) to quantify colocalization of GNPs (red) with late endosome (green) upon application of ACs. A value of 1 represents perfect colocalization of Texas red (GNPs) with the green channel (late-endosome).

We next tested our hypothesis that HF-AC affects subcellular structures involved in intracellular trafficking by studying the endosomal escape of GNPs in primary heterogeneous brain tumor cell cultures after the treatment with HF-AC (Figure 4). HF-AC (1–5 MHz at 1 V/cm) was able to induce endosomal escape of GNPs from primary glioma cells isolated from the invasive edge viz. GIN cells (Figure 4a) and the tumor core, viz., GCE cells (Figure 4b). No significant toxicity was observed after GNP, AC, and GNP + AC treatment (Figure S3). This was further validated by obtaining Pearson’s correlation coefficient (PCC) which predicted the degree of overlap between green channel (late endosomes) and GNPs (red).^27,28^ The low values of the PCC obtained from confocal microscopy images shown in Figure 4a,b confirmed that application of HF-AC leads to endosomal escape of GNPs. The low Pearson correlation coefficient (PCC) scores in your images could indicate a lower degree of colocalization between the gold nanoparticles (GNPs) and the targeted cell structures, suggesting that the nanoparticles may not be efficiently associating with or entering the desired cellular compartments. This could result from factors like the nanoparticles not reaching or penetrating the target cells effectively, the AC frequency not optimally disrupting the endosomal membranes to release the nanoparticles, or possibly the GNPs aggregating or forming nonuniform distributions that do not overlap well with the cellular markers. These factors could reduce the efficacy of the intended therapeutic action of the GNPs.

This could be explained based on previous studies that suggest that HF-AC can induce transmembrane potentials, which cause transient disruption in cell membrane structures, without causing appreciable toxicity. Importantly, it has been reported that low MHz frequencies can penetrate deep into the cytoplasm to manipulate subcellular structures.^29^ Based on these studies, the possibility of GNP escape from endosomes cannot be ruled out; however, leaking of 100 nm GNPs due to the electro-permeabilization of endosomal membrane requires further studies. Another possible mechanism that could be considered is the behavior of GNPs as electric field transducers.^19^ In this case, the polarization of GNPs in the presence of AC could allow them to interact with the plasma membrane in a way that facilitates their transport across. However, it is unclear how HF-AC could manipulate endosomal membrane, thus highlighting the need for new investigations on understanding the underlying mechanism such as the AC-mediated proton sponge effect. In the literature, there are various reports about the use of external stimuli, such as light and ultrasound, to enhance the cytoplasmic concentration of drugs from polymeric or metallic nanoparticles traversing outside the endosomal compartment.^30,31^ However, it must be emphasized that this effect relies on the properties of the conjugating ligand or peptides that facilitate endosomal escape but not on the external stimuli. Although conjugation of ligands that enhance endosomal escape has shown great potential, they have been criticized for causing offset toxicity and reducing the surface coverage for the attachment of targeting moieties.^31,32^ On the other hand, this work highlights the potential of using external electrical stimuli, such as AC, to enhance the cytoplasmic concentration of not only small-molecular-weight drugs but also metallic nanoparticles.

The application of HF-AC represents a promising strategy to enhance the therapeutic efficacy of nanoparticle-based drug delivery systems by facilitating endosomal escape. The exact mechanism has yet to be elucidated. However, we tentatively suggest that HF-AC can induce transmembrane potentials, leading to temporary disruptions in the lipid bilayers of cellular membranes, including endosomal membranes. This electroporation effect increases the permeability of the endosomal membrane, enabling the release of entrapped therapeutic agents, such as GNPs into the cytoplasm. Furthermore, GNPs, when polarized by AC, may act as transducers that interact with cellular membranes, promoting their escape from the endosomal compartments. These mechanisms collectively contribute to an increased intracellular concentration of therapeutic agents, which is crucial for overcoming the degradation limitations associated with endosomal entrapment.

Additionally, electric fields have been shown to modulate drug resistance mechanisms in cancer cells by disrupting EV pathways responsible for drug efflux. Cancer cells often utilize EVs to expel chemotherapeutic drugs, thereby enhancing their resistance to treatment. The application of AC can compromise the integrity of these vesicles, reducing drug efflux and consequently increasing the retention of drugs within the cells. By capacitive coupling with cell membranes, HF-AC can selectively target subcellular structures involved in drug trafficking, enhancing the intracellular concentration of drugs and sensitizing resistant cancer cells to chemotherapy. These effects underscore the potential of AC as a bioelectronic intervention to circumvent resistance mechanisms and improve therapeutic outcomes in aggressive tumors. This dual ability to facilitate endosomal escape and inhibit drug efflux highlights the transformative potential of combining bioelectronic approaches with conventional anticancer therapies.

## 4 Conclusions

Our findings support the hypothesis that chemotherapeutic resistance in aggressive brain tumors may be mediated via intracellular trafficking of increased numbers of EVs. Importantly, we have shown that AC can disrupt this EVs mediated trafficking of anticancer drugs out of the cell to enhance vulnerability in drug-treated medulloblastoma cells. Furthermore, we showed that HF-AC could enhance the endosomal escape of GNPs in patient-derived GBM cells. Overall, together with the ease of HF-AC delivery with no appreciable toxic effects on cells by itself, the future application of AC in drug delivery can be potentiated to achieve enhanced therapeutic efficacy for better treatment outcomes of cancer.

## Data Availability

All the data will be available to the readers at free of cost at https://nottingham.rdmc.ac.uk.

## Supporting Information Available

The data related to live dead staining of medulloblastoma cells at frequencies below <1 MHz (Figure S1), live dead images of vehicle cell lines (Figure S2), and biocompatibility of GNPs in primary GBM cells (Figure S3) (PDF)
