# Supplementary material for: Tackling Anticancer Drug Resistance and Endosomal Escape in Aggressive Brain Tumors Using Bioelectronics
Source: ACS Omega. 2024 Oct 8;9(42):42923–31. doi: 10.1021/acsomega.4c05794 (PMC11500143; doi:10.1021/acsomega.4c05794)
Supplement: Supplementary file 1 — ao4c05794_si_001.pdf [file ao4c05794_si_001.pdf]

Tackling Anticancer Drug Resistance and Endosomal Escape in Aggressive Brain Tumors Using Bioelectronics

Akhil Jain,[1,5,‡,] Philippa Wade,[2,‡] Snow Stolnik,[3] Alistair N. Hume,[4] Ian D. Kerr,[4] Beth Coyle,[2] Frankie Rawson[5,*]*

[1]*Division of Pharmacy and Optometry, School of Health Sciences, Faculty of Biology, Medicine and Health, University of Manchester, Manchester M13 9PT, U.K.*

[2]*Children's Brain Tumour Research Centre, School of Medicine, University of Nottingham, Biodiscovery Institute, Nottingham, NG7 2RD, UK*

[3]*Division of Molecular Therapeutics and Formulation, School of Pharmacy, University of Nottingham, Nottingham, NG7 2RD, UK*

[4]*School of Life Sciences, University of Nottingham, Queen's Medical Centre, Nottingham, NG7 2UH, UK*

[5]*Bioelectronics laboratory, Division of Regenerative Medicine and Cellular Therapies, School of Pharmacy, University of Nottingham, Biodiscovery Institute, Nottingham, NG7 2RD, UK*

[*]*Corresponding author email – Frankie.Rawson@nottingham.ac.uk and Akhil.Jain@manchester.ac.uk*

[‡]*These authors contributed equally.*

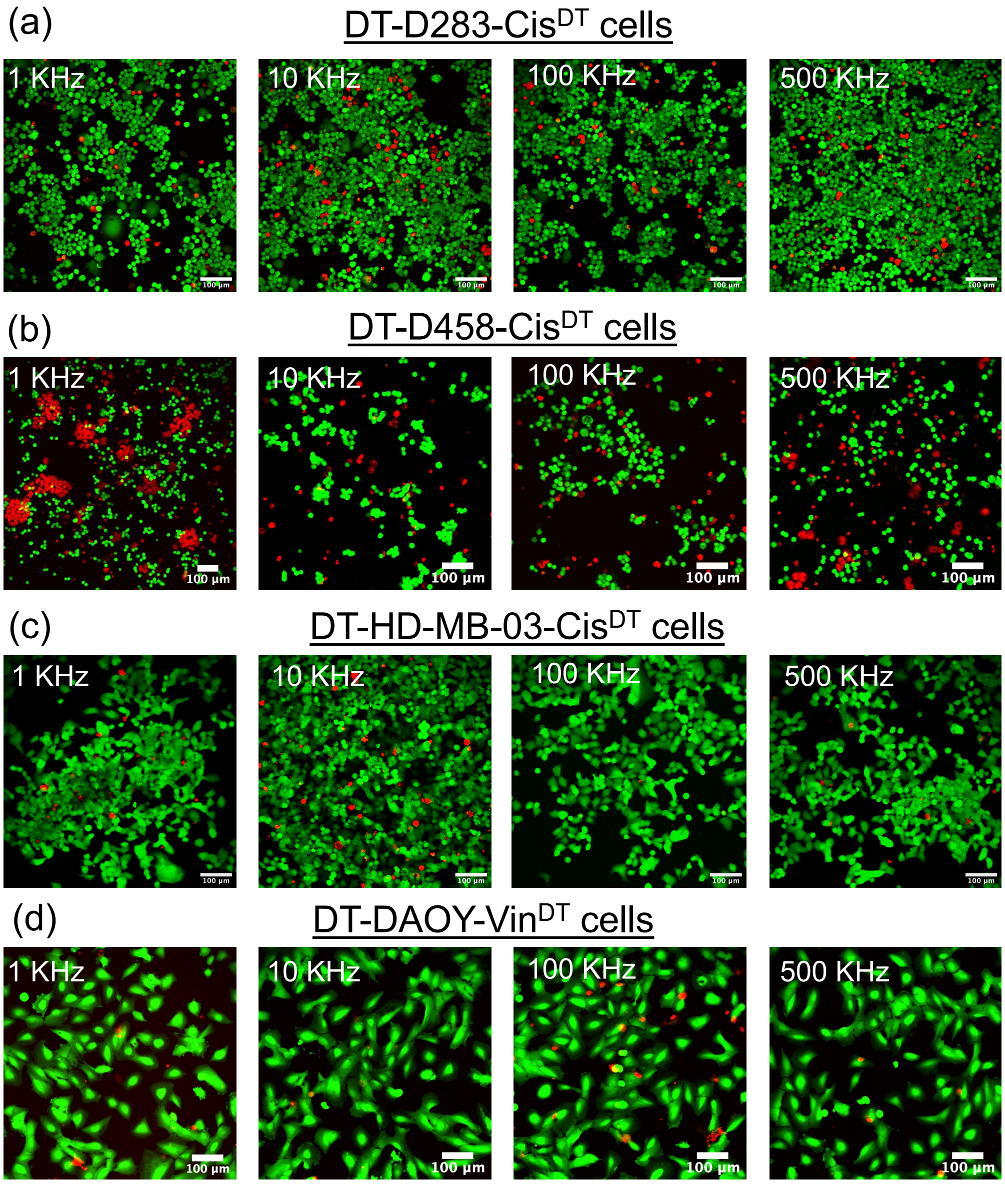

Figure S1. – AC overcomes cis and vin resistance in medulloblastoma cells *in vitro*. The cells were stimulated with square wave AC (1 kHz, 10 kHz, 100 kHz, and 500 kHz) using a frequency generator at a potential of 1V/cm for 30 min. Live/dead staining of cis and vin resistant medulloblastoma cell lines. The cells were stained with calcein AM (green, live cells) and propidium iodide (red, dead cells) 24 h after stimulation with AC and imaged using GFP and Texas red filter in a Leica TCS SPE Confocal Microscope. Scale bars = 100 µm.

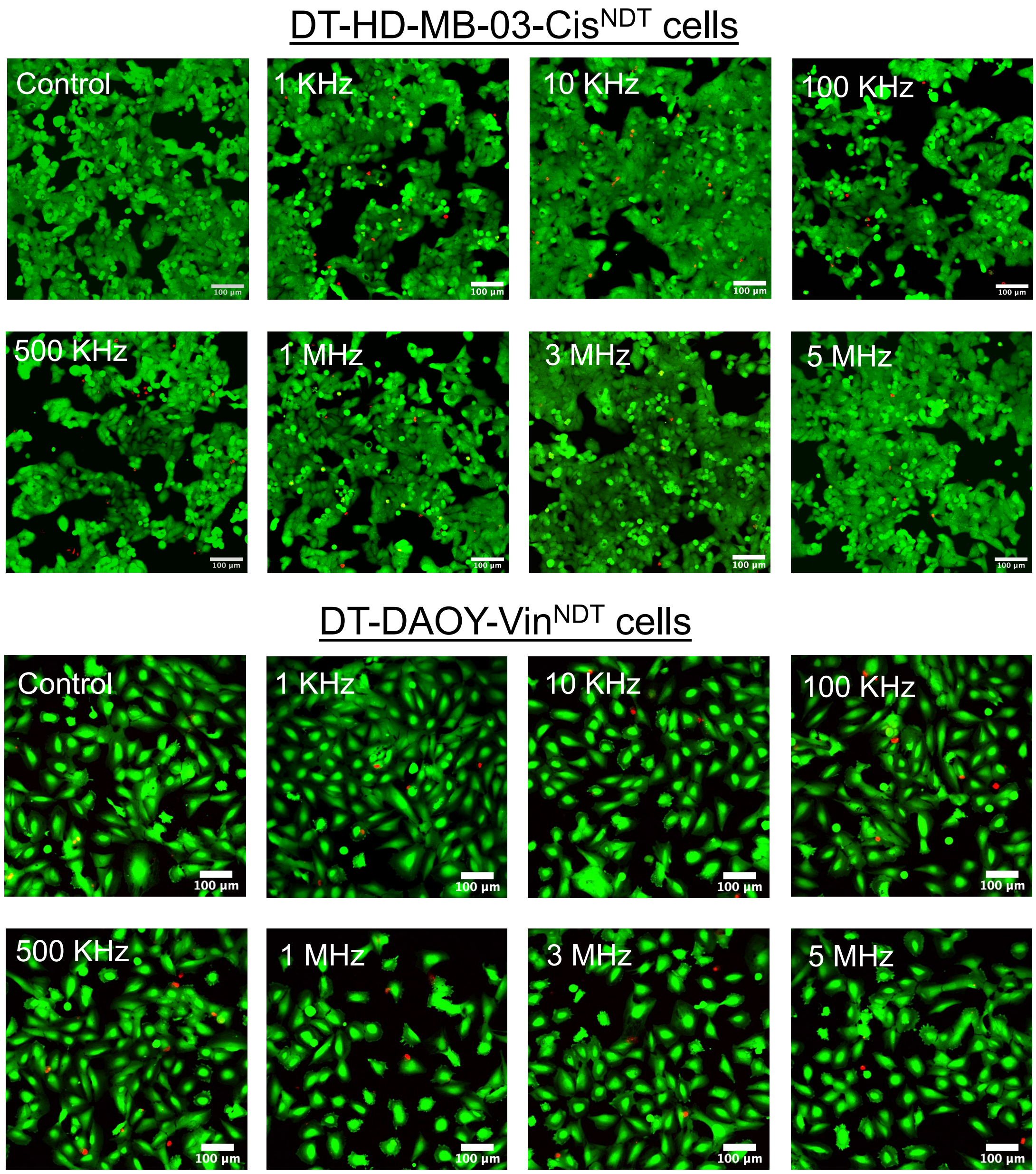

Figure S2. Live/dead staining of vehicle medulloblastoma cell lines. The cells were stained with calcein AM (green, live cells) and propidium iodide (red, dead cells) 24 h after stimulation with AC and imaged using GFP and Texas red filter in a Leica TCS SPE Confocal Microscope. Scale bars = 100 µm.

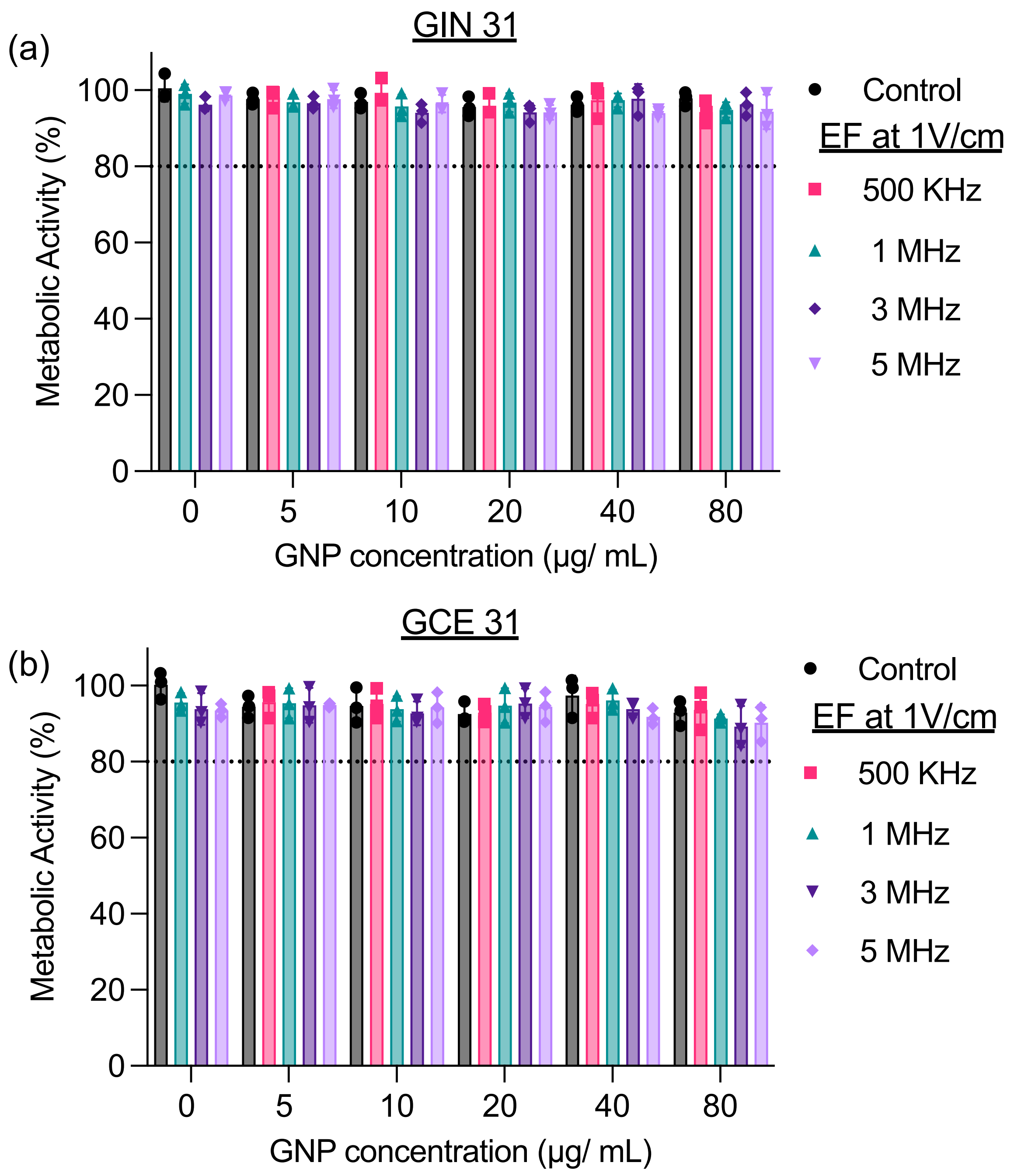

Figure S3. Biocompatibility of GNPs on GIN 31 and GCE 31 in presence of AC. The cells were treated with GNPs for 8 h before stimulation with square wave AC (1 kHz, 10 kHz, 100 kHz, and 500 kHz) using a frequency generator at a potential of 1V/cm for 30 min. The metabolic activity of cells was determined 24 hours after stimulation with AC using PrestoBlue assay. The error bars represent the S.E.M. from a triplicate experiment repeated thrice.
